# Zebrafish patient-derived xenograft models predict lymph node involvement and treatment outcome in non-small cell lung cancer

**DOI:** 10.1186/s13046-022-02280-x

**Published:** 2022-02-09

**Authors:** Zaheer Ali, Malin Vildevall, Gabriela Vazquez Rodriguez, Decky Tandiono, Ioannis Vamvakaris, Georgios Evangelou, Georgios Lolas, Konstantinos N. Syrigos, Alberto Villanueva, Michael Wick, Shenga Omar, Anna Erkstam, Julia Schueler, Anna Fahlgren, Lasse D. Jensen

**Affiliations:** 1 BioReperia AB, Linköping, Sweden; 2 Pathology Department, Athens Chest Hospital “Sotiria”, Athens, Greece; 3 3rd Department of Internal Medicine and Laboratory, National & Kapodistrian University of Athens, Athens, Greece; 4 InCELLiA P.C, Athens, Greece; 5 Program Against Cancer Therapeutic Resistance (ProCURE), Catalan Institute of Oncology (ICO), Bellvitge Institute for Biomedical Research (IDIBELL), Oncobell Program, L’Hospitalet del Llobregat, Barcelona, Catalonia Spain; 6 Xenopat S.L., Parc Cientific de Barcelona (PCB), Barcelona, Spain; 7 XenoSTART, San Antonio, TX USA; 8 Division of Cardiovascular Medicine, Department of Medical and Health Sciences, Linköping University, Campus US, Entrance 68, Pl. 08, SE-58185 Linköping, Sweden; 9 Charles River Laboratories, Freiburg, Germany; 10 Division of Cell Biology, Department of Biomedical and Clinical Sciences, Linköping University, Linöping, Sweden

**Keywords:** Cancer, Zebrafish, Metastasis, Dissemination, Drug response, Xenograft, Lymph node, PDX, ZTX

## Abstract

**Background:**

Accurate predictions of tumor dissemination risks and medical treatment outcomes are critical to personalize therapy. Patient-derived xenograft (PDX) models in mice have demonstrated high accuracy in predicting therapeutic outcomes, but methods for predicting tumor invasiveness and early stages of vascular/lymphatic dissemination are still lacking. Here we show that a zebrafish tumor xenograft (ZTX) platform based on implantation of PDX tissue fragments recapitulate both treatment outcome and tumor invasiveness/dissemination in patients, within an assay time of only 3 days.

**Methods:**

Using a panel of 39 non-small cell lung cancer PDX models, we developed a combined mouse-zebrafish PDX platform based on direct implantation of cryopreserved PDX tissue fragments into zebrafish embryos, without the need for pre-culturing or expansion. Clinical proof-of-principle was established by direct implantation of tumor samples from four patients.

**Results:**

The resulting ZTX models responded to Erlotinib and Paclitaxel, with similar potency as in mouse-PDX models and the patients themselves, and resistant tumors similarly failed to respond to these drugs in the ZTX system. Drug response was coupled to elevated expression of EGFR, Mdm2, Ptch1 and Tsc1 (Erlotinib), or Nras and Ptch1 (Paclitaxel) and reduced expression of Egfr, Erbb2 and Foxa (Paclitaxel). Importantly, ZTX models retained the invasive phenotypes of the tumors and predicted lymph node involvement of the patients with 91% sensitivity and 62% specificity, which was superior to clinically used tests. The biopsies from all four patient tested implanted successfully, and treatment outcome and dissemination were quantified for all patients in only 3 days.

**Conclusions:**

We conclude that the ZTX platform provide a fast, accurate, and clinically relevant system for evaluation of treatment outcome and invasion/dissemination of PDX models, providing an attractive platform for combined mouse-zebrafish PDX trials and personalized medicine.

**Supplementary Information:**

The online version contains supplementary material available at 10.1186/s13046-022-02280-x.

## Background

Cancer patients exhibit highly individual variations in treatment outcome and tumor invasiveness, the cause of which are poorly understood [1]. These variations complicate effective personalized treatment planning, which in turn is important for ensuring successful treatment outcomes. Surgical resection of tumors is often considered the most effective treatment option for patients with in situ or minimally invasive disease, but should be avoided in patients with more widely disseminated disease [1]. Patients with Stage 2 cancer where local invasion of tumor cells into surrounding tissues is associated with an increased risk of tumor dissemination to the lymph nodes, can be considered either for direct surgery [1] or for neoadjuvant (i.e. pre-surgical) chemotherapy. The aim of neoadjuvant chemotherapy is to down-stage/shrink the tumor prior to surgery and eradicate small, undetectable micro-metastatic lesions, thereby increasing the chances of durable remission [2]. As such, medical treatment planning depends on an understanding of the extent of tumor dissemination.

Currently, diagnostic principles for determining invasiveness and dissemination of tumors rely on the tumor-node-metastasis (TNM) staging principle. The N or M parameters, e.g. the spread of cancer to the lymph nodes or metastasis to distal organs, can however only be determined from medical imaging which require lesions of one centimeter in diameter or larger [3, 4]. These techniques therefore only diagnose late-stage (node positive or metastatic) disease rather than early-stage tumor dissemination that often lead to recurrence and progression after surgery. The gold-standard for determining invasiveness is to stage the tumor itself (e.g. the “T” in the TNM principle) by evaluating its size and local invasiveness from medical imaging and/or examination of biopsies [5]. In addition, tumor biopsies can be evaluated for expression of epithelial-to-mesenchymal transition (EMT) markers, leading to assessment of an EMT-score [6] and the differentiation status of the tumor cells. As tumors that are more mesenchymal-like and/or considered to be less differentiated may be more invasive, these diagnostic principles are currently used to help oncologists evaluate the likelihood of tumor dissemination to lymph nodes or peripheral organs. Both T-staging, EMT scores and tumor differentiation evaluations, however, suffer from poor diagnostic sensitivity and specificity, and more predictive methods for evaluating tumor invasion and dissemination are therefore urgently needed.

After a decision has been made to provide medical treatment to a patient, oncologists need to decide which among several approved treatments that are most likely to efficiently induce regression of patient tumor(s). Currently, the gold standard for predicting treatment outcome is to use patient-derived xenograft (PDX) models generated in immunocompromised mice [7–9]. Functional drug response in such models have been shown to be much more accurate predictors of response in the patients compared to for example genetic or other biomarker analyses [10, 11], and mouse-PDX models are therefore popular tools in basic cancer research and for assessing inter-individual variation to new drug candidates during (late) pre-clinical drug development. Because of these strengths, large repositories containing thousands of unique PDX models have been generated [10, 12, 13]. These models are often highly characterized including thorough analyses of mutational and transcriptional profiles, biomarker expression profiles, sensitivity to standard of care drugs, EMT score, tumor differentiation, and clinical data (including TNM staging, and demographical data). As such, PDX-models have become highly valuable resources for pre-clinical studies. Local and systemic dissemination is however not accurately evaluated using PDX models as these are generally not found to seed lymph node metastases regardless of whether they come from patients with lymph node positive or negative disease. As such alternative methods are needed whereby information regarding tumor dissemination and the anti-metastatic efficacy of drugs can be derived and coupled to the wealth of other data associated with existing PDX models.

Recently, zebrafish tumor xenograft (ZTX) systems have emerged as a powerful complementary in vivo system for research in oncology and tumor biology [14, 15]. Generating ZTX models by implantation of tumor cell lines, sometimes in combination with non-malignant tumor-infiltrating cells to model the tumor microenvironment, has led to a much deeper understanding of the molecular and pathophysiological events that drive early tumor dissemination [16, 17], crosstalk within the tumor microenvironment [18, 19] and response to drugs or immune therapies [20–22]. As established cell lines, however, may drift phenotypically over time [23], these models are mainly used for basic research or early pre-clinical drug development. To better recapitulate patient-specific cancer, ZTX models have been generated from direct implantation of non-expanded patient tumor samples and shown to accurately recapitulate the drug response profiles of the corresponding patients to both medical treatments [24–28] and radiation [29]. Tumor biopsies used for such tests are, however, newly acquired and have therefore not been characterized to the extent of established cell lines or PDX models. They are furthermore small and as they are not expanded in the ZTX models, they can only be used for one or a few studies, after which the patient sample has been consumed. Exploiting cryopreserved PDX models as the starting material in the ZTX platform could potentially overcome these issues but methods to combine PDX and ZTX models are currently lacking. Furthermore, it is currently not known to what extent the resulting ZTX models would retain important pathophysiological characteristics of the patients cancer, such as drug response and tumor dissemination profiles.

Lung cancer has the highest mortality of all cancer types [30]. Non-small cell lung cancer is the most common type of lung cancer accounting for approximately 85% of all cases [31]. High mortality from non-small cell lung cancer is largely due to the inefficiency of current medical treatments as they rarely endow the patients with long term remission [31], combined with a lack of diagnostic techniques for accurately determining the risk of systemic dissemination. As such there is a great interest in developing new and better drugs as well as diagnostic techniques for non-small cell lung cancer patients. This has led to the generation of a large number of non-small cell lung cancer (NSCLC) PDX models, that in turn have been heavily used and characterized [9, 32, 33]. Due to the importance of early dissemination in the prognosis of this disease, there is great interest in targeting this aspect of tumor development.

Here we develop new and robust methods for implanting NSCLC PDX material into zebrafish larvae demonstrating excellent implantation rates leading to cohorts of tumor-baring zebrafish larvae being established for 88% of the 43 models tested. The ZTX models accurately recapitulated responses to paclitaxel and erlotinib seen in the corresponding mouse-PDX models and in the patients themselves. Importantly, the ZTX platform predicted tumor dissemination to the lymph nodes of the patients with 91% sensitivity, while predicting the absence of lymph node involvement with 62% specificity. Primary patient samples also implanted efficiently providing a proof-of-principle that ZTX models can be established directly from patient biopsies without prior expansion in mice. ZTX analyses based on implantation of PDX models thereby accurately recapitulate both drug response and invasive phenotypes of non-small cell lung cancer.

## Methods

### Patient recruitment and ethics

After written informed consent, tumor tissue from NSCLC patients undergoing surgery was placed in a storage solution and transported within a few hours to Charles River, XenoSTART or Xenopat. Incoming material of every donor patient received a chronological unique number which was subsequently used to identify the corresponding PDX model. For direct use of patient biopsies as the starting material for generation of ZTX models, small tumor samples were placed in cryopreservation medium during surgery, cryopreserved and shipped on dry ice to BioReperia. The patients were anonymized by prospective numbering. Ethical approval for studies based on primary tumor biopsies were granted to K. Syrigos by the regional ethical committee.

### Reagents

Collagenase (Cat #C5138), hyaluronidase (Cat #H6254), NaCl (Cat #S5886), CaCl_2_ (Cat #C8106) were purchased from Sigma. DNAse (Cat #11284932001) from Roche. Dispase (Cat #17105–041) from Gibco. RPMI-1640 culture medium (Cat #LM-R1637/500) from Biosera. Fetal bovine serum (Cat #97068–085), MgSO4 (Cat #0662) and KCl (Cat #26764.232) from VWR. Gentle MACS™ C-tube (Cat #130096334) from Miltenyi Biotec. Trypan blue 0.4% solution (Cat #15250061) from ThermoFisher Scientific. Erlotinib (Cat #S7786), and paclitaxel (Cat #S1150) from Selleckchem. 1-Phenyl-2-Thiourea (aka PTU, Cat #L06690) from Alfa Aesar.

### Generation and banking of mouse PDX models

This study was carried out in strict accordance with the recommendations in the Guide for the Care and Use of Laboratory Animals of the Society of Laboratory Animals (GV SOLAS). All animal experiments were approved by the Committee on the Ethics of Animal Experiments of the regional council (Permit Numbers: G-09/58, G-13/13 and G13/43). 4–6 week old female NMRI nu/nu mice, bred in house, were placed under isoflurane anesthesia and received tumor implants subcutaneously in one or both flanks. During the first passages, mice were monitored for tumor growth for up to 12 months. When stable tumor growth could be determined, mice were sacrificed and tumor material was implanted into new recipient mice. Commonly 4–5 mice were used for each passage. In addition, xenograft material was stored in liquid nitrogen for future implantation or fixed in formalin for subsequent analyses.

### Characterization of mouse PDX models in vivo (doubling time, take rate, etc)

A PDX was defined as established when stable growth over at least three and often more than five passages and regrowth from cultures stored in liquid nitrogen could be observed. As 4–5 mice were used for each model and passage, a total of 12–25 were used to establish a model. The percentage of tumor implants displaying stable growth (take rate) and passage time were recorded across at least three independent experiments (*n* = 25 for each experiment) for every model. Tumor growth was determined by a two-dimensional measurement with calipers weekly or biweekly depending on the growth characteristics of the respective PDX model. Tumor volumes were calculated according to the following equation: Tumor Vol [mm^3^] = a [mm] x b2 [mm^2^] × 0.5, where “a” is the largest diameter and “b” is the perpendicular diameter of the tumor representing an idealized ellipsoid. Animals had to be sacrificed when tumor volume reached 1.800 mm^3^. The metadata of the donor patient and corresponding PDX are listed in Supplemental Table 1. For mouse-PDX model drug-treatment experiments, 5 mice were used per model and treatment group (including non-treated controls).

### Histological characterization of mouse PDX models

Tumors were collected immediately after euthanasia of the donor animal. FFPE fixation was performed in 10% neutral buffered formalin for 24 h followed by routine processing and embedding into paraffin. H&E stains of all samples were performed.

### Genomic and transcriptomic analysis of PDX models

#### Whole exome sequencing

After DNA extraction, material was sequenced with 126 bp paired-end reads using the ILLUMINA HiSeq-2500 platform and the Agilent V5 50MB enrichment kit, with a coverage of >160X. Next, human reads were isolated using Xenome [34], aligned to the GRCh38 reference genome, and mutations were called using a workflow based on GATK (Genome Analysis Toolkit version 4) best practices. Using the variant effect predictor (VEP) [35], candidate mutations were annotated and filtered considering only variants with moderate or high protein impact and those being rare in healthy populations (< 1% in gnomAD) [36]. Furthermore, mutations shared by all three resistant clones were annotated with protein functions using SIFT and Polyphen predictions from SNPnexus [37].

#### Affymetrix HGU133plus2

Gene expression profiles were obtained by Affymetrix HGU133plus2 microarray chips and subjected to stringent quality control measures. Expression values were extracted from. CEL files, calculated and log 2 transformed using R/Bioconductor packages (gcRMA).

#### RNAseq

Total RNA was digested by DnaseI (NEB) and purified by oligo-dT beads (Dynabeads mRNA purification kit, Invitrogen). Then poly(A)-containing mRNA were fragmented into 200-250 bp with Fragment buffer (Ambion). Sequencing was done using Illumina HiSeq 2000/2500/4000 in 100/126 bp paired-end (PE) reads with an expected throughput of 10G bases per sample. Next, mouse stroma was discarded and reads were utilized to obtain gene expression (using tophat2 and cufflinks), to call mRNA mutations (following the GATK best practice pipeline), to identify fusion genes (using fusioncatcher), and to decipher the HLA-type (using seq2HLA).

### Assessment of tumor EMT-scores

The EMT score was determined based on the gene set published by Taube et al. [6]. It describes the expression level of an EMT score signature based on RNAseq data of 14 NSCLC PDX models described in this study.

### Zebrafish strains, maintenance, and ethics

The zebrafish strains used in this study were raised and maintained in the zebrafish facility at Linköping University, Linköping, Sweden as described [38]. Tg (fli1a:EGFP)^y1^ fish [39] were purchased from ZIRC (Oregon). All experiments were approved by Linköping animal research ethical committee. Zebrafish embryos were collected and maintained in E3 embryo medium (containing 0.286 g NaCl, 0.048 g CaCl_2_, 0.081 g MgSO_4_ and 0.0126 g KCl per liter, pH 7.2) supplemented with 0.2 mM PTU (E3/PTU) at 28.5 °C.

### Implantation of PDX models into zebrafish larvae and drug treatment

Tumor tissue samples were thawed and washed gently by inversion with 10 mL of RPMI-1640 medium supplemented with 10% FBS (RPMI/10% FBS), then, the tissue samples were minced using surgical scissors to obtain ~ 1–3 mm^3^ pieces. Five milliliters of PDX-disruptor Mix (Cat #P01–3001, BioReperia, Sweden) were added to the minced tissue and transferred to a Gentle MACS™ C-tube and dissociated in a gentleMACS™ Octo Dissociator for 30–60 min at 37 °C. The resulting single cell suspension was washed with RPMI/10% FBS, filtered and finally labeled with 8 μg/mL Fast-DiI™ oil (ThermoFisher Scientific Cat #D3899) in RPMI/2% FBS for 30 min at 37 °C. Cells were then washed, re-filtered and viable + non-viable, trypan blue positive cells were counted.

DiI-labeled cells were implanted subcutaneously in the perivitelline space of 2 days old zebrafish embryos as previously described [40]. Correctly injected embryos (embryos without injected cells in the yolk or in circulation) were selected under a fluorescent stereoscope model M205 FA (Leica Microsystems CMS GmbH) and primary tumors were photographed using the Leica Application Suite X (LAS X) software v3.7.1.21655 (Leica Microsystems CMS GmbH). Embryos were randomly sorted into experimental groups of 20 embryos per group and incubated during 72 h at 36 °C with E3/PTU water containing erlotinib 10 mg/L, paclitaxel 20 mg/L or vehicle. Incubation at 36 °C did not lead to any toxic phenotypes or reduced survival compared to incubation at 28.5 °C and was chosen over lower temperatures to provide the best growth conditions for the human tumor implants. After incubation, tumor bearing-embryos were photographed using the LAS X software and pictures of primary tumors and the caudal venous plexus were obtained.

### Analysis of tumor growth regression and dissemination in zebrafish larvae

Images were acquired using a fluorescent stereoscope (Leica) equipped with a K5 camera (Leica) with resolution (h x v) 2048 × 2048 pixels and pixel size (h x v) 6.5 × 6.5 μm, at an image magnification of 100x with no binning. Images were obtained right after implantation (0 days post injection, dpi) and after 72 h incubation (3 dpi) and analyzed by using the HuginMunin software v2.7.0.0 (BioReperia AB, Linköping, Sweden). Tumor sizes were analyzed as the area of labeled tumor cells at 0 dpi and 3 dpi, and the number of disseminated cells to the caudal venous plexus were manually counted. Relative tumor size was calculated by dividing the tumor area at 3 dpi by the area at 0 dpi in the same embryo and multiply by 100.

### Drug toxicity assay in zebrafish

Zebrafish embryos were collected and maintained in E3/PTU at 28.5 °C for 48 h. At 2 days post-fertilization, embryos were randomly separated into treatment groups of 20 embryos per group. Treatment of 1, 10 and 100 mg/L erlotinib and 5, 20 and 80 mg/L paclitaxel was added in the E3/PTU water and embryos were incubated for 72 h at 36 °C. Parameters such as living embryos, pericardial edema, head and tail necrosis, malformation of head and tail, teratogenesis, brain haemorrhage and yolk sack edema were recorded every 24 h for 72 h.

### Statistics

Results were generated in a blinded fashion. Binominal distribution was confirmed using the Kolmogorov-Smirnov test and data is shown as means +/− SEM from representative experiments. Statistical comparisons were made using two-tailed, unpaired students t-test assuming equal variance between the groups. n-values represent the number of individual larvae analyzed. All statistical analyses were performed and generated using Prism 7 (GraphPad Software).

## Results

### Cryopreserved PDX tissue fragments from mouse-PDX models implant robustly in zebrafish embryos

**Fig. 1:**
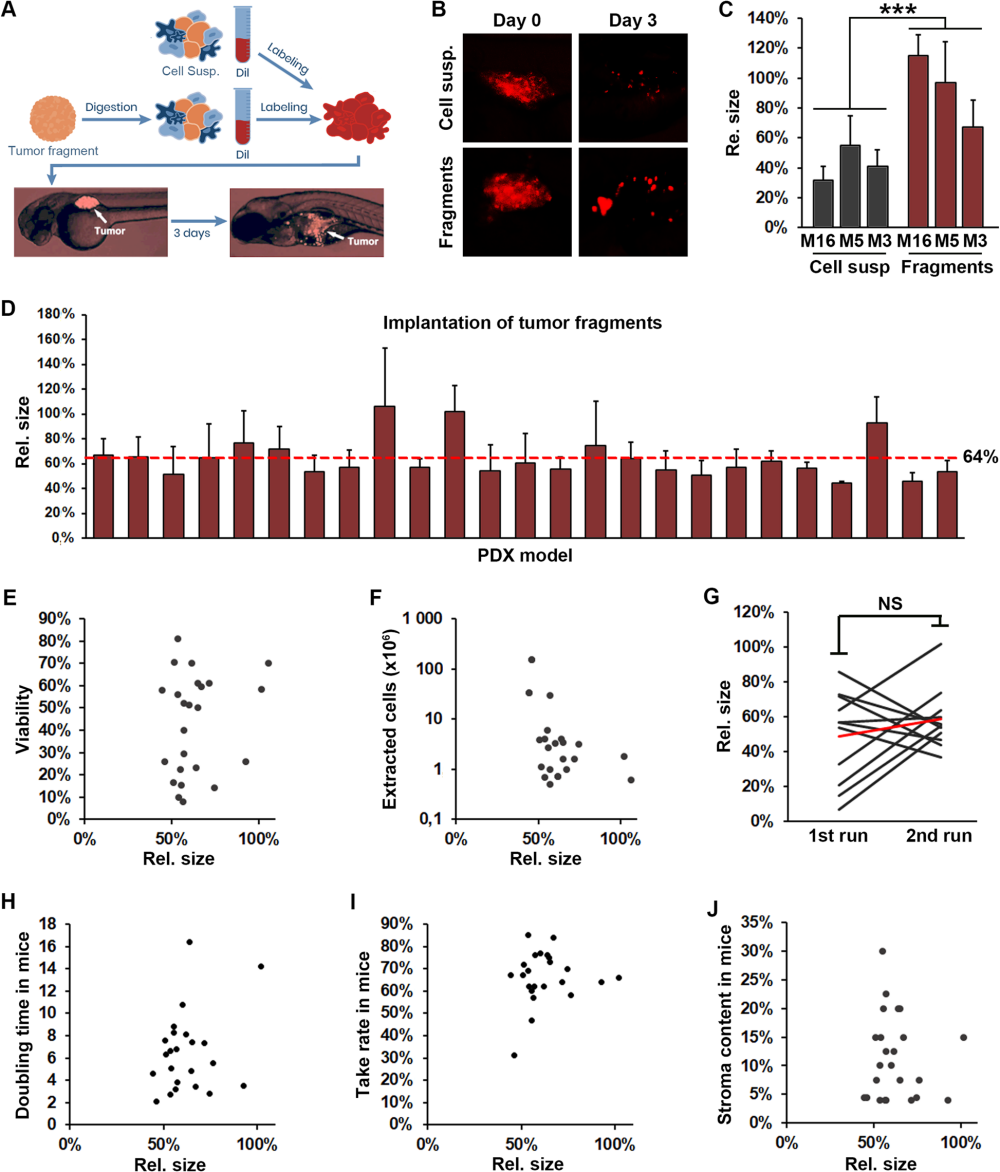
Robust implantation and growth of NSCLC PDX models in zebrafish larvae. **A** Cartoon illustrating the tissue handling, implantation and visualization of tissue fragments or cell suspensions from mouse PDX material into zebrafish larvae. **B** Representative fluorescent micrographs of DiI-labeled xenografts (shown in red) generated from cell suspensions (top row) or tissue fragments (lower row), imaged immediately and three days after implantation (left and right columns respectively). **C** Quantifications of changes in tumor size (rel. size) between day three and zero for three separate models (M16, M5 and M3) that were implanted either as fragments or as cell suspensions in separate cohorts of zebrafish larvae. *n* = 18, 15, 8 larvae in the cell suspension groups and 17, 18, 14 larvae in the fragments groups respectively. *** = *p* < 0.001. **D** Quantifications of changes in tumor size (rel. size) between day three and zero for the 25 models that was implantable in the zebrafish larvae. Average size-change was 64% for all models combined. *n* = 12–33. **E**,**F** Quantification of changes in tumor size (rel. size) between day three and zero plotted against the viability (**E**) or number of cells extracted from the fragments (**F**). *P* > 0.05. **G** Quantification of changes in tumor size (rel. size) between day three and zero for 11 models run at two different times. Red line indicates the average of the 11 models. NS: non-significant. **H-J**: Quantifications of changes in tumor size (rel. size) in zebrafish larvae between day three and zero plotted against the tumor doubling time (**H**), take rate (**I**) or stromal content (**J**) when growing subcutaneously in mice. *P* > 0.05

To establish methods for generating zebrafish tumor xenograft (ZTX) models, we obtained 30 extensively characterized non-small cell lung cancer models from the Charles River PDX model repository, 6 models from the XenoSTART PDX model repository and 3 models from Xenopat’s PDX model repository (Supplemental Table 1). The models were selected to span three major histotypes including similar numbers of adenocarcinomas, squamous cell carcinomas and large cell carcinomas. The samples were derived from a representative mix of approximately 2/3 males and 1/3 females and at different TNM-stages upon diagnosis including 13 without lymph node involvement, 20 with lymph node involvement and 6 where such information was not available (Supplemental Table 1). The histological characteristics of the models were similar to those of the primary patient tumors even after serial passaging (Supplemental Fig. S1) indicating that the significant heterogeneity observed between the patients was retained in the cryopreserved models. As most of these models are available both as cryopreserved cell suspensions and cryopreserved tissue fragments, we first investigated which of these resources would lead to the best implantation rate of the models in zebrafish larvae. Prior to implantation, cryopreserved tissue fragments were enzymatically and mechanically disaggregated and compared head-to-head against cryopreserved cell suspensions from the same models. After disaggregation or thawing respectively, the cells were labelled with the fluorescent dye DiI, their viability was determined, and finally the labeled cells were implanted subcutaneously in the perivitelline space of 2-days old zebrafish larvae (Fig. 1A). Images were taken of each tumor baring larvae immediately after implantation and again 3 days later, and the relative change in tumor size was determined (Fig. 1B and C). In all three head-to-head comparisons, cells derived from cryopreserved tissue fragments, that were disaggregated after thawing, gave rise to more viable tumors compared to if the tissues were disaggregated first, cryopreserved as cell suspensions and then thawed (Fig. 1C). On the average tumors grew 2.2 fold larger relative to their size at implantation in the tissue fragments group compared to the cell suspensions group. We therefore decided to use cryopreserved tissue fragments disaggregated after thawing, in all future experiments.

Next, we evaluated the implantation efficiency in the zebrafish larvae. Among the thirty Charles River models, 25 allowed generation of a sufficiently large number of tumor baring embryos (*n* > 20), which was taken as the criteria for successful implantation. For the 5 that did not implant this was due to either too few cells resulting from the disaggregation process (< 10^5^, *n* = 3), or cells aggregating to an extent that did not permit injection into the larvae (*n* = 2). The 25 models that implanted resulted in tumors that at 3 dpi were on average 64% of the size at implantation (Fig. 1D), suggesting that tumor cell death was in general largely balanced by tumor proliferation during the 3 days growth period. Interestingly, the relative tumor size at 3 dpi did not correlate with cell viability prior to implantation for most of the models tested (Fig. 1E). As dead cells are generally much smaller (e.g. apoptotic bodies), we hypothesize that these may be excluded from the viable cell fraction during cell preparation prior to implantation or when the cells sediment in the injection needle during the implantation procedure. This could explain why dead cells might not contribute to a significant extent to the implanted tumor in the ZTX model and therefore why growth is not dependent on the amount of dead cells found in the sample prior to implantation. The relative tumor size of three models that generated highly proliferating tumors (rel. Size ≥100%) did, however, exhibit a trend towards more robust growth in models having higher cell viability prior to implantation (Fig. 1E). Similarly, relative tumor size also did not correlate with the number of cells that could be extracted from the sample (Fig. 1F).

Next, to establish the robustness of the ZTX platform, eleven models were re-run using separate vials of cryopreserved tissue fragments, thawed and processed on separate days, by a different investigator. While relative tumor sizes at 3 dpi varied slightly for some models, the ZTX tumor development did not differ overall between the first and second run (Fig. 1G), indicating that the ZTX platform is robust.

We also evaluated two important parameters of tumor development and growth in mice, tumor doubling time and take rate, of all thirty models, and compared these to the growth of the corresponding ZTX models. Similar to the ZTX models, the mouse PDX-models demonstrated variable growth and take rates (Fig. 1H and I), that, however, correlated poorly with the relative growth of the corresponding ZTX tumors. As some tumors have high stroma content, and as non-malignant stroma cells could be less proliferative compared to the tumor cells and thereby contribute less to tumor growth in the days following implantation, we investigated whether high stroma content could be the coupled to smaller relative tumor sizes in the models. Again, whereas stroma content in the models varied between 4 and 30% (Supplemental Fig. S1), this did not correlate with the relative size of the ZTX tumors at 3 dpi (Fig. 1J).

### Treatment responses to standard of care drugs are accurately recapitulated using ZTX models

**Fig. 2:**
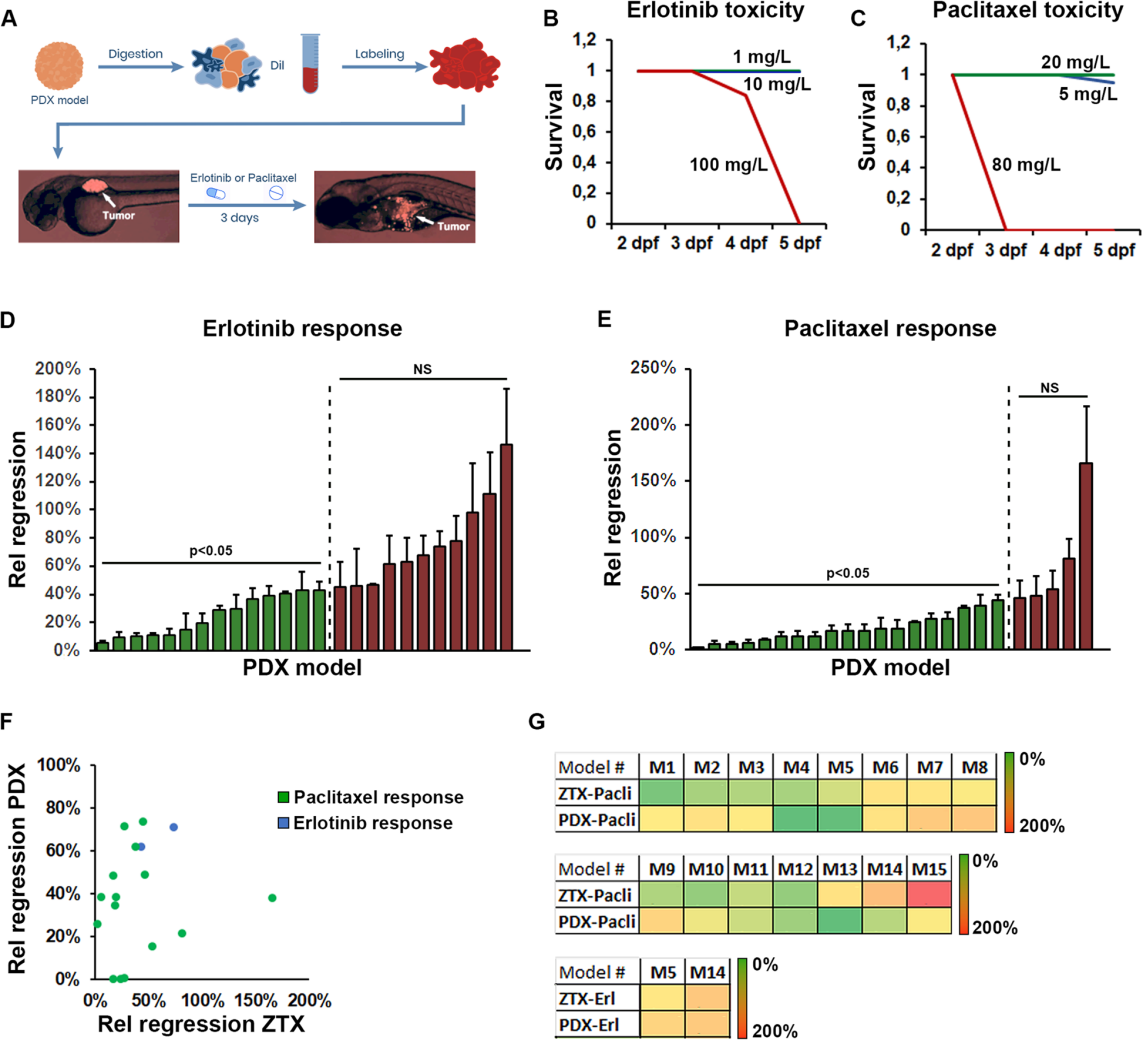
Similar response to erlotinib and paclitaxel are seen in ZTX and mouse PDX models. **A** Cartoon illustrating the tissue handling, implantation, and visualization of tissue fragments from mouse PDX material into zebrafish larvae followed by 3-days treatment with Erlotinib or Paclitaxel added to the water. **B-C** Quantification of the proportion of surviving embryos following treatment starting at 2-days post fertilization with the indicated concentrations of Erlotinib (**B**) or Paclitaxel (**C**) for one, two or three days. *n* = 20 (**B**) and *n* = 18 (**C**) per group. Blue lines indicate 1 and 5 mg/L, green lines indicate 10 and 20 mg/L and red lines indicate 100 and 80 mg/L in **B** and **C** respectively. **D-E** Quantification of the changes in tumor size between day three and zero for larvae treated with either Erlotinib, 10 mg/L (**D**) or Paclitaxel, 20 mg/L (**E**), divided by the changes in tumor size between day three and zero for larvae in the corresponding control groups (Rel regression) for each of the 25 models tested. Models where treatment led to significant tumor regression are shown in green whereas models where the drug did not significantly induced regression is shown in red. *n* = 7–22 per group, NS: non-significant. **F** Quantification and correlation of the treatment outcome for Erlotinib (blue dots) or Paclitaxel (green dots) plotted as relative treatment-induced regression in mouse-PDX models against that of the relative treatment-induced regression in the corresponding ZTX models. **G** Heat map of the degree of relative regression as quantified in **D** and **E** and shown in **F** for zebrafish tumor xenograft (ZTX) models and in a similar manner when PDX models grew in mice PDX, for each of the 15 models in which drug efficacy was tested in both zebrafish and mice. Erl: Erlotinib, Pacli: Paclitaxel

Next, we investigated drug sensitivity and resistance profiles of the initial cohort of 25 implantable Charles River models in the ZTX platform and compared these to the corresponding profiles of the mouse-PDX models. We focused on two commonly used drugs for non-small cell lung cancer, Paclitaxel, a classic cytotoxic agent and Erlotinib, a targeted inhibitor of the EGF receptor. These drugs were added to the water immediately after tumor implantation and thus present during the entire three-day incubation period (Fig. 2A). As the clinically relevant doses for these drugs are 20 mg/kg and 10 mg/kg for Paclitaxel and Erlotinib respectively, we first tested whether these doses would lead to unwanted side-effects in the developing zebrafish larvae. Giving the drugs to healthy, non-tumor baring 2-days old larvae and incubating them at 36 degrees (the permissive temperature for both tumor growth and survival of the larvae), we found that both Paclitaxel and Erlotinib were tolerated at the targeted concentrations (Fig. 2B and C), although toxicity could be observed at higher doses. While we found one dead embryo in the group treated with the lower dose of 5 mg/L Paclitaxel, we considered that this embryo likely died from other causes (e.g. hatching failure or other developmental defects), and not from Paclitaxel toxicity, as no embryos were found dead at 20 mg/L.

We next treated 20 tumor baring larvae for each of the 25 models with either vehicle, Erlotinib or Paclitaxel and evaluated the relative tumor size in each group at 3 dpi compared to 0 dpi. Normalizing the relative size of tumors in the drug-treated groups to that of tumors in the vehicle-treated control group we obtained a measure of relative drug-induced regression for the models (Fig. 2D and E). Erlotinib and Paclitaxel effectively caused primary tumor regression in 14 of 25 and 20 of 25 models respectively, although these drugs were much more potent in some models compared to others (Fig. 2D and E). These results might reflect the broader mechanism of action of Paclitaxel compared to Erlotinib and demonstrate the highly variable individual responses of different PDX models to standard of care treatments.

We next chose 15 models at random and determined the drug response profiles to Erlotinib and Paclitaxel in mice for 2 and 15 of these respectively. The models were chosen to represent all three histotypes (7 adenocarcinoma, 5 squamous cell carcinoma and 3 large cell carcinoma), to recapitulate the heterogeneity of the disease. As expected Erlotinib and Paclitaxel inhibited tumor growth in mice with different potencies ranging from complete inhibition (tumors were undetectable and therefore at 0% of vehicle-controls at the endpoint) to 73.7% inhibition (Fig. 2F and G). Importantly, the efficacy of these drugs in the ZTX models correlated very well with their tumor growth inhibitory efficacy in the mouse-PDX models (Fig. 2F and G). Only in one case a PDX model exhibited accelerated growth upon Paclitaxel treatment (166% of the relative tumor size in the non-treated group) in the ZTX model, whereas this tumor exhibited Paclitaxel-induced regression to 38% of the tumor size in the control group, in the corresponding mouse-PDX experiment. Overall, however, the ZTX platform accurately recapitulate responses to standard of care treatments of non-small cell lung cancer PDX models.

### Paclitaxel or Erlotinib efficacy could be epi-genetically but not genetically determined

**Fig. 3:**
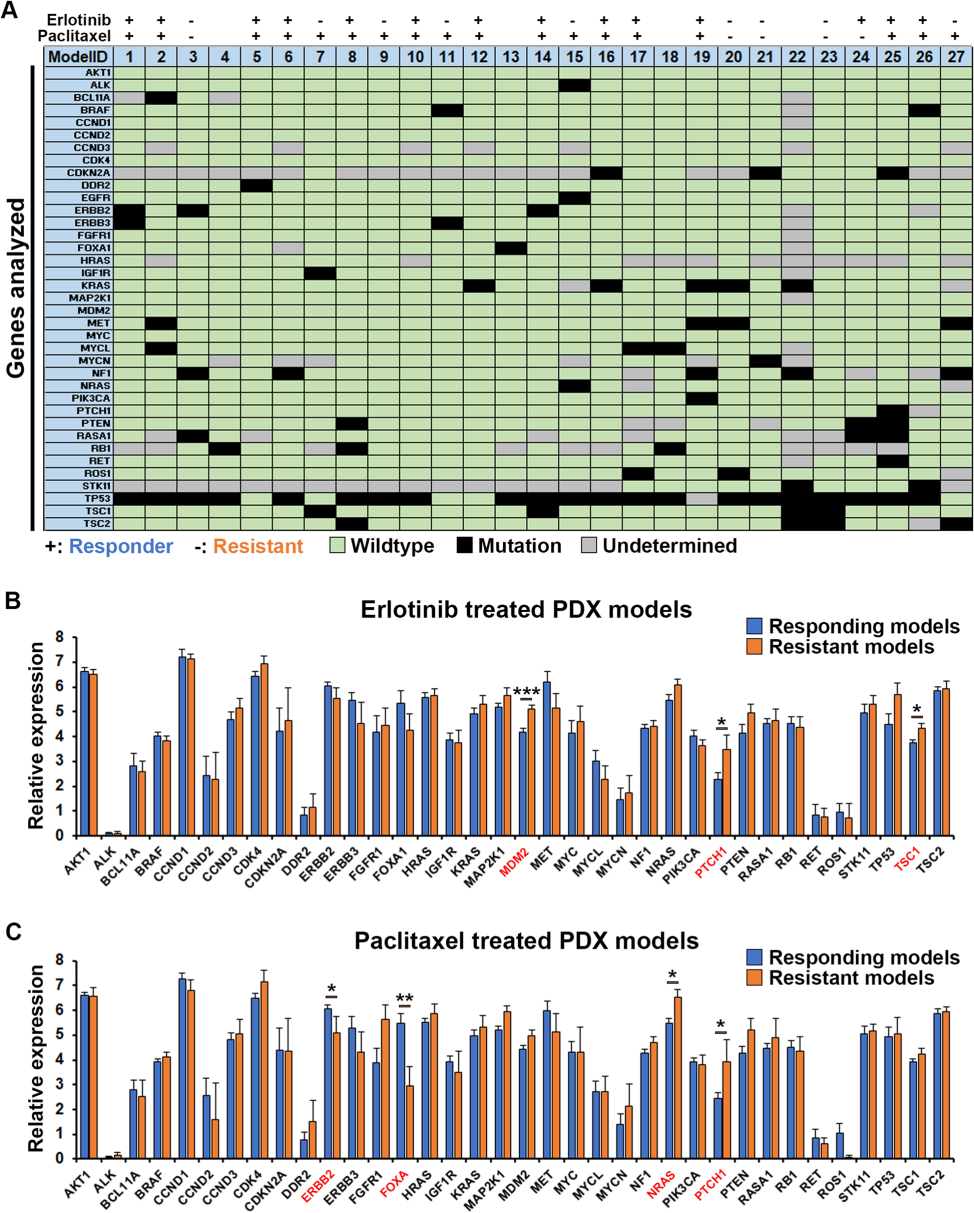
Genetics of tumors and correlation with treatment outcome. **A** Scheme showing the genotype of the 37 genes included in this study against the 27 PDX models analyzed. All types of mutations were combined and indicated as black squares, whereas the absence of a mutation is indicated as wildtype in green. Where low coverage did not allow an accurate assessment, this is indicated in grey. Response or lack of response to Erlotinib or Paclitaxel in the ZTX models is indicated with + or – respectively above the scheme. **B** Average relative expression of the 36 genes included in this study (EGFR is shown in Supplemental Fig. S2) as evaluated by RNA sequencing, for models responding (*n* = 14 models, blue bars) or resistant (*n* = 9 models, orange bars) to Erlotinib. *:*p* < 0.05, ***:*p* < 0.001. **C** Average relative expression of the 36 genes included in this study (EGFR is shown in Supplemental Fig. S2) as evaluated by RNA sequencing, for models responding (*n* = 18 models, blue bars) or resistant (*n* = 5 models, orange bars) to Paclitaxel. *:*p* < 0.05, **:*p* < 0.01

Next, to investigate whether certain mutations or changes in gene-expression levels could be predictive of response versus resistance to Erlotinib or Paclitaxel, we analyzed the profile of mutations and expression levels of 37 genes commonly associated with cancer biology and pathology (Fig. 3A-C). Using next generation sequencing we identified various types of mutations including splice donor, −acceptor, or -region variants, coding sequence variants, point mutations, point mutations that led to gain of a stop codon, frameshift mutations, intron mutations or in-frame deletions (Supplemental Table 2). Grouping all these different types of mutations we found that most of the genes analyzed were wildtype in the majority of the models, with the exception of tp53 that was mutated in 21 of 27 models. Some genes, however, were only partially analyzed due to poor coverage and therefore have undetermined mutational status in some of the models. Comparing the mutational profiles to the drug response profiles of 27 models, we found no clear pattern of mutations conferring sensitivity or resistance to neither Erlotinib nor Paclitaxel (Fig. 3A). Interestingly, the only model exhibiting a mutation in the Egfr-gene, which is often used to indicate Erlotinib treatment, was found to be resistant to Erlotinib in the ZTX model. It is now recognized, that certain, “non-classical” Egfr mutations are associated with resistance rather than response to Erlotinib [41–43], which could potentially explain these findings. In addition to EGFR-mutation, overexpression may also be used as a biomarker for Erlotinib sensitivity [44]. We therefore specifically investigated whether response to Erlotinib in the ZTX model system depended on the EGFR-expression level for 19 of the models tested, using gene-expression data from the Charles River PDX model compendium. Both when EGFR-expression was evaluated using HGU133 microarrays and when using RNA-sequencing, 17 of the 19 models demonstrated a robust correlation between EGFR-expression level and relative Erlotinib-induced tumor regression (Supplemental Fig. S2). These results provide a likely explanation for why some of the EGFR-wildtype tumors responded to Erlotinib treatment.

We further examined the expression level of an additional 36 genes included in this panel by RNA-sequencing (Fig. 3B and C). Comparing the expression in sensitive models to that of resistant models, we identified that over-expression of the P53-inhibitory factor Mdm2, the sonic hedgehog receptor Patched1 (Ptch1) and the mTOR-inhibitor TSC1 was associated with resistance to Erlotinib (Fig. 3B). Similarly, overexpression of Patched1 but also of NRAS was associated with resistance to Paclitaxel, whereas overexpression of ERBB2 and FOX-A was significantly associated with Paclitaxel response (Fig. 3C). These results suggest that high expression of Patched1 might confer resistance to first line treatment in lung cancer, a hypothesis that should be further investigated in future clinical studies.

### Drug-induced inhibition of tumor dissemination is not correlated with anti-tumor efficacy

**Fig. 4:**
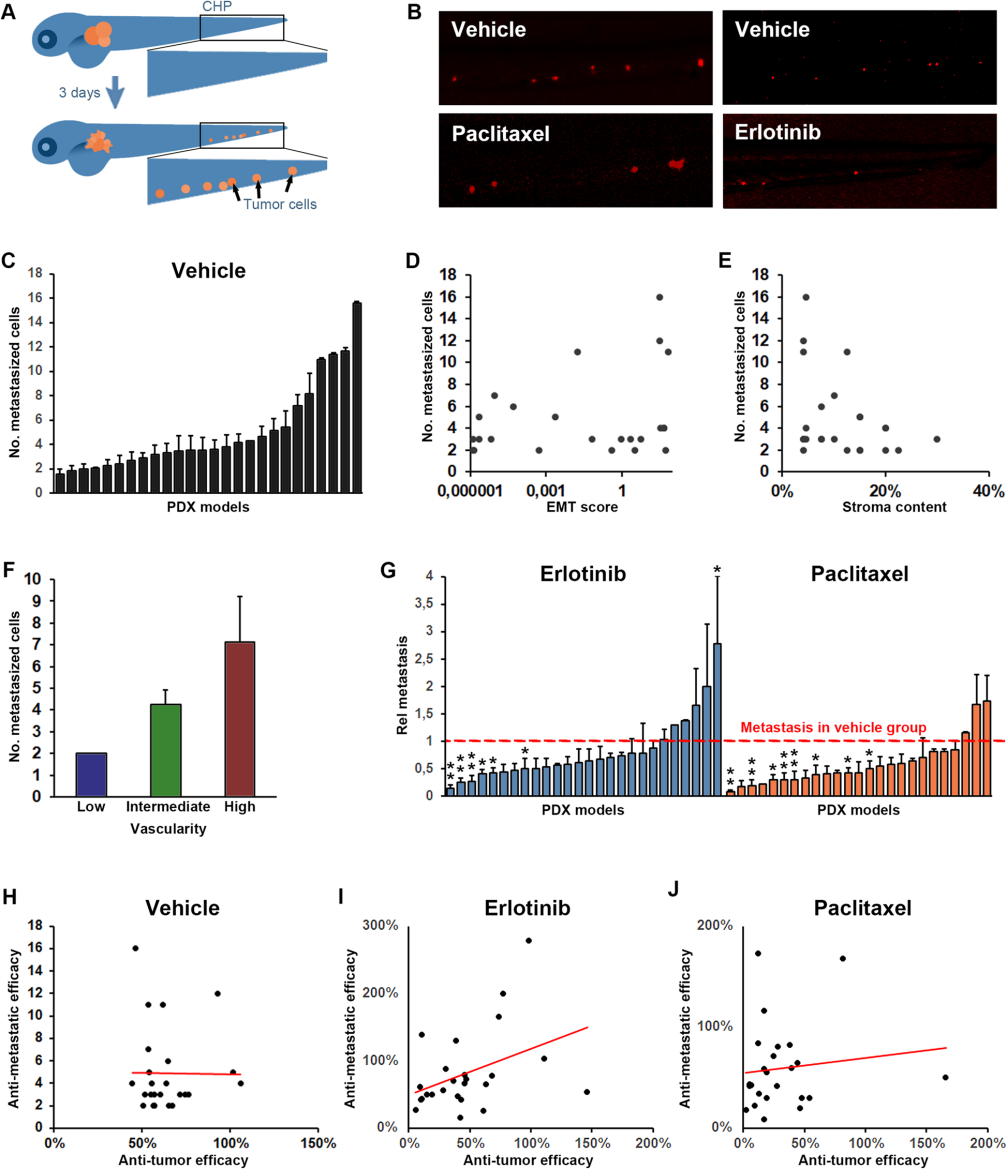
Tumor dissemination correlates with vascularity of the tumor but not tumor growth/regression rate. **A** Cartoon illustrating dissemination of tumor cells (shown in orange) to the caudal hematopoietic plexus (marked by the black squares) three days following tumor implantation in the perivitelline space. **B** Representative images of tumor cells (shown in red) in the caudal hematopoietic plexus three days after tumor implantation for a representative model in which dissemination was moderately but significantly inhibited by treatment with Erlotinib (10 mg/L, right panels) but not by treatment with Paclitaxel (20 mg/L, left panels). **C** Quantification of the average number of cells that at three days post implantation have disseminated to the caudal hematopoietic plexus (metastasized cells) for each of the implantable models. **D-F** Quantification of the EMT-score (**D**), stromal content (**E**) and vascularity (**F**) of the PDX models when grown in mice plotted against the average number of metastasized cells, as quantified in **C**, three days after implantation of the models in zebrafish larvae. Positive correlation was significant for metastasis and vascularity but not for metastasis and EMT-score or stromal content. *n* = 12–33 per group. **G** Quantification of the relative change in metastasis to the caudal hematopoietic plexus in treatment compared to vehicle groups after treatment with either Erlotinib (10 mg/L, shown in light blue), or Paclitaxel (20 mg/L, shown in orange). Red dashed line indicates the control group for each model. *n* = 7–22 per group. **H-J** Quantification of the changes in tumor size between day three and zero for larvae in the control group (**H**) or after treatment with Erlotinib, 10 mg/L (**I**), or Paclitaxel, 20 mg/L (**J**), divided by the changes in tumor size between day three and zero for larvae in the corresponding control groups (anti-tumor efficacy) and plotted against the relative change in metastasis as quantified in **G**, for each of the 25 implantable models. A non-significant trend towards a positive correlation was observed in the Erlotinib treated group but not in the Paclitaxel treated groups. *n* = 7–22 per group

One of the major benefits of ZTX models is the possibility to evaluate early tumor invasion and dissemination. To investigate if the ZTX platform could also determine invasiveness and dissemination of patients tumors using PDX models, we implanted zebrafish larvae with cells generated from the initial set of 25 implantable PDX models, and removed larvae where cells had been erroneously transferred to circulation during the implantation process. The number of labeled tumor cells that had emerged in the main metastatic niche at the caudal hematopoietic plexus was subsequently evaluated at 3 dpi (Fig. 4A-C). As expected, the models exhibited highly heterogeneous metastatic capabilities in the ZTX-system (Fig. 4C), which provided an opportunity to investigate diagnostic or prognostic factors associated with invasion. We therefore determined, based on gene-expression and histopathological analyses, to what extent factors previously coupled to invasiveness, including the EMT-score, stromal content, and vascularity of the tumors (Supplemental Fig. S1), might correlate with the ability of the models to seed metastatic cells in the zebrafish larvae. Whereas the EMT-score and stromal content of the models did not correlate with the number of metastatic cells seeded in the zebrafish (Fig. 3D and E), higher vascularization of the PDX models prior to implantation in the zebrafish larvae was clearly associated with augmented seeding of metastatic cells (Fig. 4F), suggesting that the angiogenic properties of a model is more closely coupled to their metastatic dissemination compared to the EMT-score or stromal content.

Next, we evaluated the anti-metastatic efficacy of Erlotinib and Paclitaxel by evaluating the metastatic response following treatment for each of the implantable models. While the treatments inhibited metastatic dissemination in some of the models (Fig. 4B and G), they were ineffective in this aspect in the majority of the 26 models tested. To evaluate whether anti-metastatic efficacy correlated with the ability of the drugs to cause primary tumor regression in the ZTX platform, we plotted the change in tumor size against the number of metastasized cells in the vehicle group (Fig. 4H) as well as the anti-tumor efficacy against the anti-dissemination efficacy for Erlotinib or Paclitaxel treated models (Fig. 4 I and J respectively). The metastatic potency was unrelated to its growth as there were no correlation between tumor growth and metastatic rate of the models (Fig. 4H). Surprisingly, however, the potency of drug-induced tumor regression was not robustly associated with potency of drug-induced inhibition of tumor dissemination for either of the two drugs tested (Fig. 4I and J), although a trend towards such a correlation could be seen from models treated with Erlotinib (Fig. 4I). This suggests that EGFR-blockade may to a larger extent inhibit both tumor growth and dissemination in sensitive models whereas the effects on tumor growth and dissemination seem to be uncoupled from each other in Paclitaxel treated models.

### Tumor dissemination in ZTX models is highly predictive of tumor dissemination in patients

**Fig. 5:**
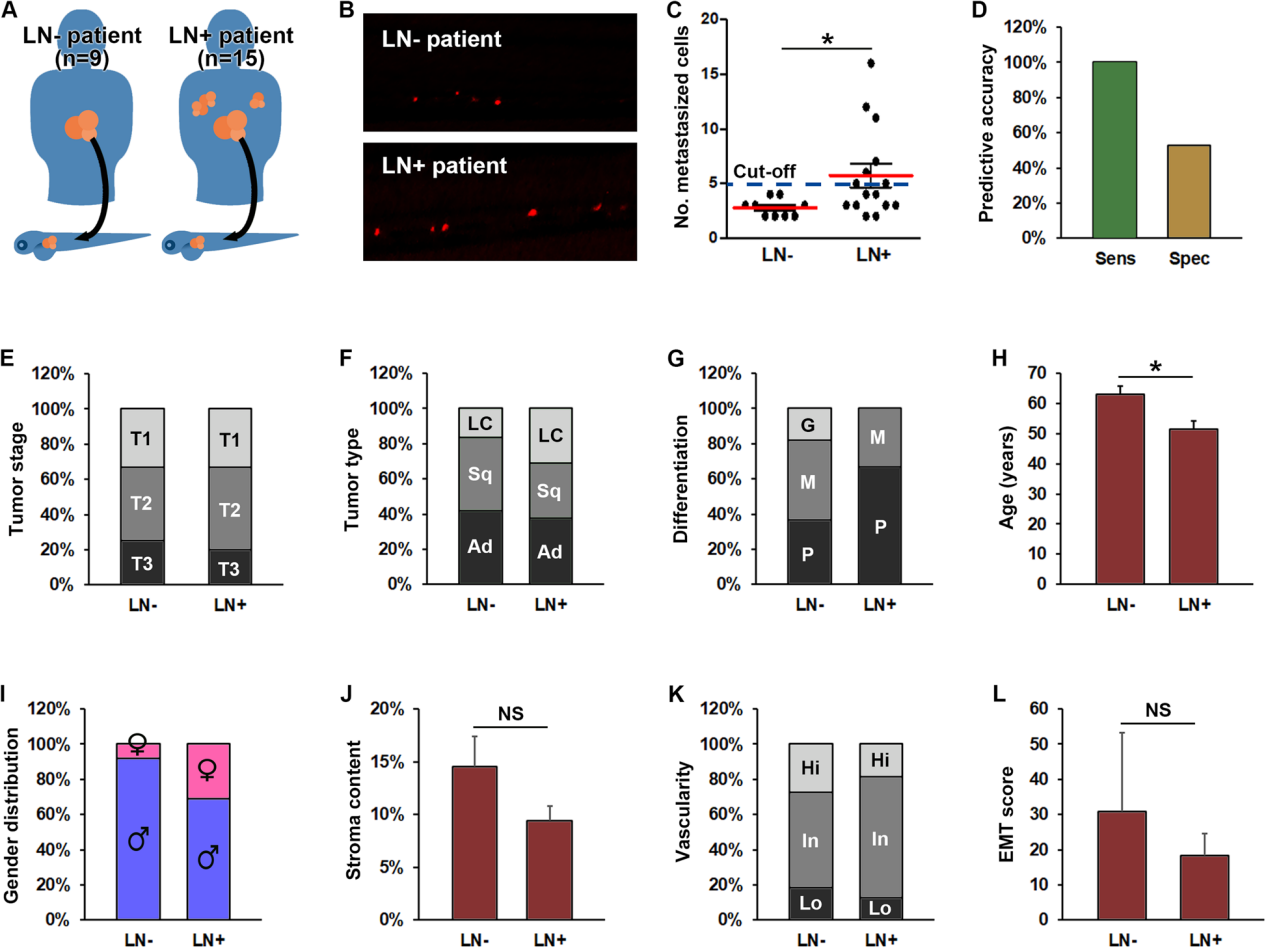
Increased metastasis in ZTX models accurately predicts lymph node involvement in the patients. **A** Cartoon illustrating implantation of PDX models generated from non-small cell lung cancer patients with or without lymph node involvement (LN+, right part of the figure and LN-, left part of the figure respectively) in zebrafish larvae. **B** Representative fluorescent micrographs of tumor cells (shown in red) disseminated to the caudal hematopoietic plexus three days after implantation from patients having lymph node negative (LN-) or positive (LN+) disease upon diagnosis. **C** Quantification of the average number of tumor cells disseminated to the caudal hematopoietic plexus (metastasized cells) three days following implantation of PDX models generated from patients with lymph node negative (LN-) or positive (LN+) disease. Dashed line indicates the establish diagnostic cut-off of 5 disseminated cells. *: *p* < 0.05. *n* = 12–33 larvae per model. **D** Quantification of the diagnostic sensitivity (accuracy of predicting patients having lymph node positive disease) and selectivity (accuracy of predicting patients not having lymph node positive disease) using the cut-off value shown in **C**. *n* = 7 and 17 for sensitivity and specificity respectively. **E** Distribution of tumor stages associated with lymph node negative (LN-) or positive (LN+) disease. No stage was significantly associated with lymph node involvement. The number of patients associated with each group is given in Supplemental Table 1. **F-I** distribution of histological tumor sub-types (**F**), tumor differentiation stages (**G**), average age (**H**), and gender (**I**), among patients with lymph node negative (LN-) or positive (LN+) disease upon diagnosis. Female gender, younger age, large cell carcinoma and poorly differentiated cancer were significantly associated with higher risk of lymph node positive disease (*p* < 0.05). The number of patients in each group is given in Supplemental Table 1. LC: Large cell carcinoma. Sq: Squamous cell carcinoma. Ad: Adenocarcinoma. **J-L** Quantification of the average stromal content (**J**), vascularity (**K**) and EMT-score (**L**) of PDX models grown in mice derived from patients with either lymph node negative (LN-) or positive (LN+) disease upon diagnosis. n-values for each group are given in **A**. NS: non-significant. G: Good. M: Moderate. P: Poor. Hi: High. In: Intermediate. Lo: Low

We next investigated to what extent the ZTX models recapitulated the invasive tumor phenotypes of the patients from which the models were derived. Of the 30 Charles River models used in the initial study, 12 came from patients with lymph node negative (LN-, aka N0) and 16 from patients with lymph node positive disease (LN+, aka N1–3) at the time of biopsy, with such information lacking from the remaining two patients (Fig. 5A). Of the 25 models that implanted in zebrafish larvae, 15 were derived from LN+ patients and 9 from LN- patients, with one coming from a patient with unknown lymph node status. Interestingly, the PDX models derived from the LN+ patients seeded on the average 2.1 fold more metastatic cells in the ZTX platform compared to models derived from LN- patients (Fig. 5B and C), an increase that was statistically significant. More importantly, of the seven models seeding 5 or more metastatic cells in the ZTX platform, all seven were derived from LN+ patients implying that lymph node involvement could be positively predicted with a sensitivity of 100% at this cut-off value (Fig. 5C and D). Furthermore, of the 17 models that seeded less than 5 metastatic cells 9 were derived from LN- patients (all LN- patient were included in this group), leading to a 53% diagnostic specificity at this cut-off value. For comparison, the best clinical standard, i.e. tumor (T)-staging, did not correlate with lymph node involvement in this patient cohort (Fig. 5E). Other, but in all cases weaker indicators of lymph-node involvement included patients diagnosed with large cell carcinoma compared to adeno- or squamous cell carcinoma (Fig. 5F) and having a moderately or poorly differentiated tumors (Fig. 5G), being of a younger age at the time of diagnosis (Fig. 5H) and being of female gender (Fig. 5I). The invasive characteristics of the PDX models in mice, including the stromal content, vascularity and EMT score, however, was not significantly different among LN+ compared to LN- patients (Fig. 5 J-L). As such, the best standard currently in clinical use for predicting lymph node involvement in this PDX-cohort was tumor differentiation status, which predicted lymph node involvement in 10 of 15 patients (sensitivity of 67%) and the absence of lymph node involvement in 7 of 12 patients (specificity of 58%) with the cut-off set at poor differentiation status (Fig. 5G and Supplemental Table 1). Using 5 metastasized cells as the cut-off, the ZTX-test exhibited a sensitivity of 100% and specificity of 53% - which is significantly more accurate compared to the differentiation predictor. Combined, these findings strongly suggest that ZTX models generated from cryopreserved PDX material closely recapitulate invasive phenotypes of the patient tumors and predict lymph node involvement with higher sensitivity than the current clinical gold standard.

### Drug responses from ZTX models is highly predictive of drug responses in patients

**Fig. 6:**
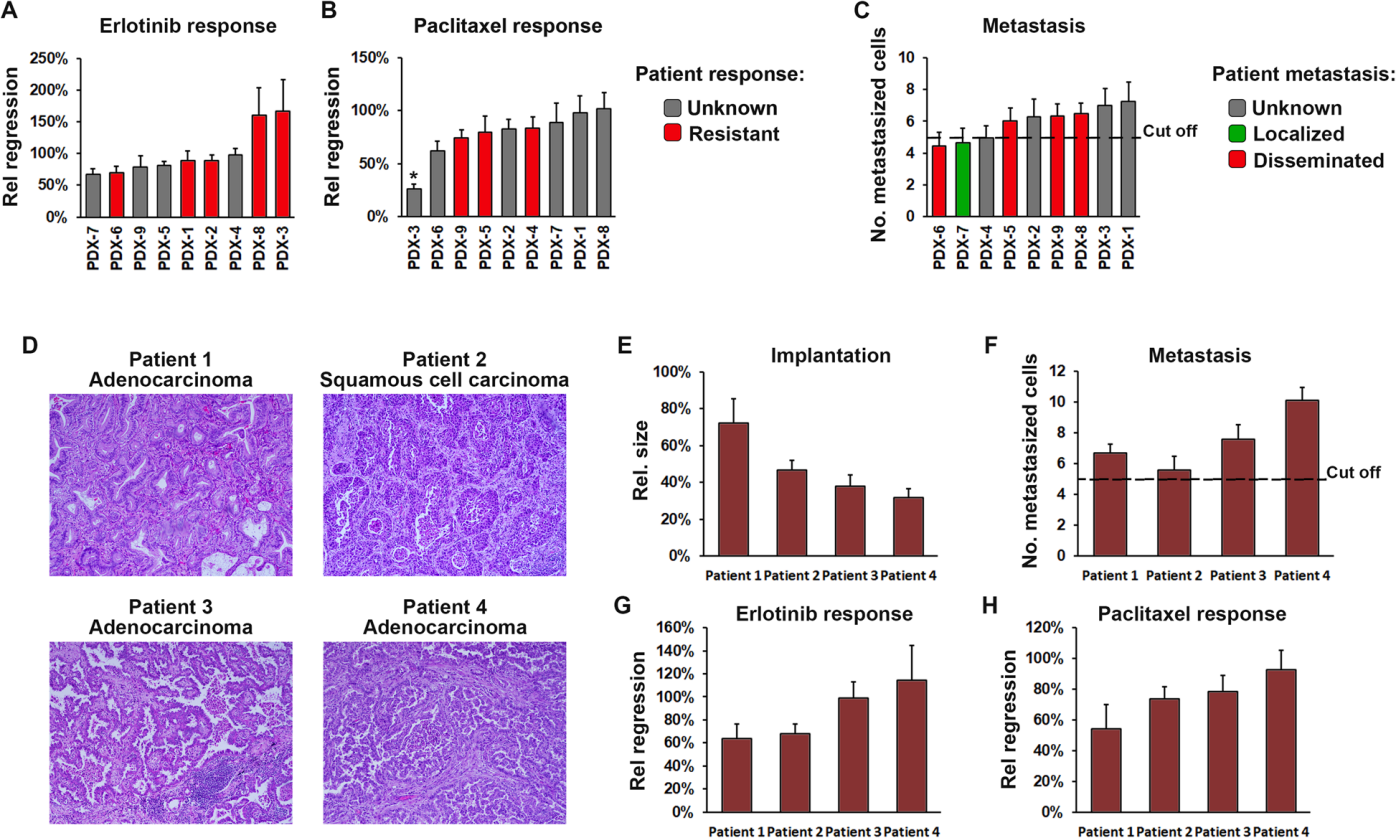
ZTX models can be established directly from patient samples and accurately predict patient treatment outcomes. **A**,**B** Quantification of the relative treatment-induced tumor regression of tumors established from 9 PDX models calculated as the change in tumor size between day 3 and 0 after implantation, in groups treated with Erlotinib 10 mg/L (**A**) or Paclitaxel 20 mg/L (**B**), divided by the change in tumor size in the control group. *n* = 13–19. Gray bars indicate models from patients that were not treated with the indicated drug and red bars indicate models from patients that had progressed following treatment with the indicated drug. *: *p* < 0.05. **C** Quantification of the average number of metastasized cells at three days after implantation for each of the 9 validation models. *n* = 13–19. Green bar indicates a model from a patient with a localized cancer, red bars indicate models from patients with disseminated cancer and grey bars indicate models from patients with unknown lymph node or metastatic status. Black dashed line indicates the cut-off level for prediction of disseminated disease by the ZTX model. **D** H&E micrographs of tumor sections from the four patients included in this study. **E-H** Quantification of the relative growth (**E**), average number of metastasized cells (**F**), and relative Erlotinib- or Paclitaxel-induced tumor regression (**G** and **H** respectively), calculated as in **A/B**, for the four patients included in this study. Dashed line indicates the cut-off level for predicting disseminated disease. *n* = 14–20

To further validate our findings from the initial cohort of 30 PDX models, and to further compare treatment outcomes in ZTX models to those observed in patients, we obtained an additional 6 NSCLC PDX models from XenoSTART and 3 NSCLC PDX models from Xenopat, for which the patient treatment outcome was known. All 9 PDX models implanted in the zebrafish (Fig. 6A-C) and exhibited varied responses to Erlotinib and Paclitaxel. Five models were derived from patients that had recurred following Erlotinib treatment (three of which, PDX1, 2 and 3, provided by Xenopat, did not obtain a response from the treatment and two, PDX6 and 8, provided by XenoSTART, that recurred after a period of remission). An additional three of the XenoSTART PDX models came from patients that had recurred following Paclitaxel treatment (all did not obtain a response). These eight models were similarly resistant to Erlotinib and Paclitaxel in the ZTX platform, thereby demonstrating a 100% correlation between the ZTX and patient treatment outcome (Fig. 6A and B). We next evaluated whether the previously set cut-off of 5 disseminated cells would also predict lymph node involvement or metastasis in these PDX cohorts. Of the six XenoSTART and three Xenopat models, only one came from a patient with localized cancer, and this model also exhibited below cut-off dissemination in the ZTX model. Of the four models that were derived from patients with known metastatic disease, three exhibited above cut-off dissemination whereas one did not (Fig. 6C). As such, also in this validation cohort of PDX models, dissemination in the ZTX system correctly predicted metastatic dissemination in the patients with 80% accuracy.

### ZTX models can be established directly from surgical NSCLC patient biopsies

Next, to determine whether also freshly collected, non-expanded NSCLC patient biopsies could be implanted in the zebrafish embryos, we set-up a clinical proof-of-concept study to which four patients were recruited. Three of the patients were diagnosed with adenocarcinoma whereas one was diagnosed with squamous cell carcinoma (Fig. 6D). The patients were of both sexes (two male and two female) and were 68 to 74 years old. Biopsies were collected for diagnostic purposes by the pathologist, cryopreserved on site and shipped on dry ice to the BioReperia Lab for analysis in the ZTX platform. All four patient samples implanted in the zebrafish embryos, although the relative size of the tumors at 3 days post implantation were slightly lower compared to when PDX models were used (Fig. 6E). All ZTX models exhibited above cut-off dissemination, which would indicate that the patients likely suffered from lymph node positive or metastatic disease. Unfortunately, all four ZTX models were also resistant to both Erlotinib and Paclitaxel, suggesting that the patients may not gain therapeutic benefit from these treatments. While larger, prospective studies are required to determine the accuracy of predicting treatment outcome and lymph node involvement/metastasis in NSCLC patients based on direct use of their tumor biopsies for establishing ZTX models, this proof-of-concept study demonstrates the feasibility of such an approach.

## Discussion

Dissemination of tumor cells to distal organs are closely linked to high mortality in non-small cell lung cancer as well as other malignancies. Patients diagnosed with early-stage, local disease are treated surgically with curative intent, but a majority of these patients relapse and most often with metastatic disease, indicating that the cancer had disseminated in spite of the initial node-negative diagnosis [45]. Adjuvant chemotherapy after surgery may improve survival but mainly in patients presenting clinicopathological signs of more aggressive disease [46]. As such, an accurate diagnosis of the likelihood of subclinical dissemination may be of key importance to select patients for adjuvant chemotherapy, but current methods are not sufficiently sensitive or specific to provide clinical value, especially for T1 tumors [46]. As a result, a significant number of patients are over-treated, i.e. receiving aggressive and highly toxic chemotherapy when in fact their tumors were completely removed by surgery alone, or under-treated in the case of patients undergoing surgery to remove the cancer, but are not given adjuvant chemotherapy and relapse within a short period of time. Improved diagnostic techniques that may predict local or distal dissemination could potentially reduce over- and under-treatment and thereby improve survival of early-stage NSCLC patients. Our findings provide clear evidence in favor of using ZTX models for identification of invasive phenotypes of non-small cell lung cancer. We show that ZTX models have higher sensitivity (91% when three cohorts studied in this study are combined) and similar specificity at predicting lymph node dissemination, compared to evaluating the tumor (T)-stage, tumor differentiation level and the EMT-score. This strongly suggests that ZTX models would provide superior diagnostic accuracy when evaluating disease stage and degree of tumor dissemination in NSCLC patients in the future. While we here provide a proof-of-principle based on four prospectively included patients that such as approach is feasible, further studies in larger cohorts of patients and/or PDX models, as well as in cohorts of patients with other types of tumors are needed, to further establish the applicability of the ZTX system for diagnosing disseminated or metastatic disease.

The high sensitivity and accuracy of predicting lymph node involvement in non-small cell lung cancer shown here is in line with studies done with other tumor types. ZTX techniques have for example been used to correctly predict lymph node dissemination in prospective clinical studies focusing on gastric cancer [27] or late stage neuroendocrine tumors [25] in 14 of 15 patients in total (i.e. 93% specificity). ZTX models have also been used extensively in basic, molecular cancer research for example to identify a hypoxia-induced VEGF-VEGFR2 signaling pathway that drove metastatic dissemination of fibrosarcoma cells [47], the pro-metastatic signaling by CCL2 and CCL5 between ER-positive breast cancer tumor cells and infiltrating macrophages [17], and the capability of CAFs isolated from metastatic cancers to educate non-invasive tumor cells to become metastatic [16].

The use of tumor cells lines or fresh patient tumor tissues for generating ZTX models is associated with either poor translation of the results to clinical situations or limited characterization and use of the material respectively. As ZTX models is consuming the tissues used for the experiments, it is important to couple such experiments to methods that expand the precious clinical samples, thereby allowing infinite experimentation. Using PDX material solve this issue as they retain the characteristics of the patient’s tumor, can be expanded, and are as such not limiting experimentation. We have developed methods allowing robust implantation of PDX models into zebrafish larvae and demonstrate that the responses to commonly used drugs are similar between the ZTX and mouse PDX systems as well as the patients themselves. Importantly, the individual variations seen in both tumor growth and drug response rate were maintained when analyzed as ZTX models. These important findings are strengthened by prior studies showing that ZTX models predict the outcome of FOLFOX, FOLFIRI or radiotherapy in colorectal cancer patients [24, 29], bortezomib or lenalidomide in multiple myeloma [28] or 5-FU in gastric cancer [27] with correct prediction of treatment outcome (either positive or negative) in 13 of 14 patients (i.e. 93% specificity) in total. As such, evidence is accumulating from several different research groups that ZTX models indeed are accurately predicting treatment outcome, with similar or better specificity compared to mouse-PDX models [10]. Existing and new PDX models brought onto the market are, however, often fully characterized in terms of mutational and transcriptomic status, growth rate, differentiation, vascularization, amount of stroma content, etc. which allow this wealth of information for each model to be exploited for understanding the specific traits of patient tumors that could be successfully targeted [9], and thereby give valuable insights for designing clinical trials [8]. As such, PDX models provide a better foundation for drug development and basic cancer research compared to fresh patient tumor samples. Ideally, however, the extensive amount of information associated with most PDX models should be exploited earlier during the drug development process where perhaps tens or hundreds of drug candidates are still under consideration, but mouse-PDX models would be impractical and too expensive to run for such a large amount of candidates [48]. Furthermore, adding information on the metastatic capabilities of the PDX models would allow their direct use as models to identify and develop anti-metastatic drugs, a potential that is currently under-exploited.

ZTX models, on the other hand, do not allow expansion of patient tumor material due to the small size of the embryos that does not allow recovering the tumors after a period of growth, combined with the poor growth rate seen during the short (3 days) analysis period. This drawback also implies makes it impossible to studying more advanced stages of the metastatic process in the ZTX models. In patients, regrowth of disseminated cells into metastatic nodules is a key parameter related to recurrence. ZTX models are well suited for studying the dissemination process itself, but not the regrowth phase, which, however, can be studied using PDX models. Therefore, combining ZTX and PDX models potentially provide synergism during late pre-clinical drug development or advanced studies in tumor biology, as the strengths of either system compensate for the drawbacks of the other.

## Conclusions

Highly accurate, fast, scalable, and reliable models for predicting treatment outcome and dissemination of individual patients’ cancers may revolutionize pre-clinical drug development and precision medicine. We have shown that zebrafish tumor xenograft (ZTX) models meet these needs by predicting treatment outcome with similar sensitivity as the current gold standard mouse-PDX models, and are significantly better than the current pre-clinical and clinical gold standard diagnostics at predicting lymph node involvement boasting 91% sensitivity in a trial that enrolled a total of 43 patient models. As this is the largest patient-derived ZTX model study to date, and the only study directly comparing ZTX models to mouse-PDX models, our study provides new and important evidence suggesting that ZTX models can be exploited to de-risk clinical drug development trials and improve personalized diagnosis of cancer patients in the future.

## Supplementary Information

**Additional file 1: Supplemental Figure S1.** Histological assessment of patient samples and PDX models. Histological features of the selected NSCLC PDX and corresponding patient tissue. H&E stains were prepared from FFPE samples of donor patient tissue as well as the fourth passage of PDX derived thereof. Whole slides were scanned and 10x magnification jpegs extracted of the scans (scale bar included). **Supplemental Figure S2.** EGFR gene-expression levels correlate with Erlotinib response. EGFR gene-expression measured in the microarray HGU133 (left graph) or by RNA-sequencing (right graph) plotted against the relative tumor size of Erlotinib-treated versus control ZTX models (low values indicate stronger response to Erlotinib). Blue dashed regression lines indicate logarithmic regressions based on the values within the blue dashed circles.

## Data Availability

Data is available by contacting the corresponding author at lasse.jensen@liu.se.

## Abbreviations

CAF: Carcinoma-associated fibroblast
DPI: Days post injection,
EGFR: Epithelial growth factor receptor
EMT: Epithelial-to-mesenchymal transition
ER: Estrogen receptor
LN: Lymph node
NSCLC: Non-small cell lung cancer
PDX: Patient-derived xenograft
TNM: Tumor-node-metastasis
ZTX: Zebrafish tumor xenograft.
