# Supplementary figures and images for: Zebrafish patient-derived xenograft models predict lymph node involvement and treatment outcome in non-small cell lung cancer
Source: J Exp Clin Cancer Res. 2022 Feb 9;41:58. doi: 10.1186/s13046-022-02280-x (PMC8827197; doi:10.1186/s13046-022-02280-x)

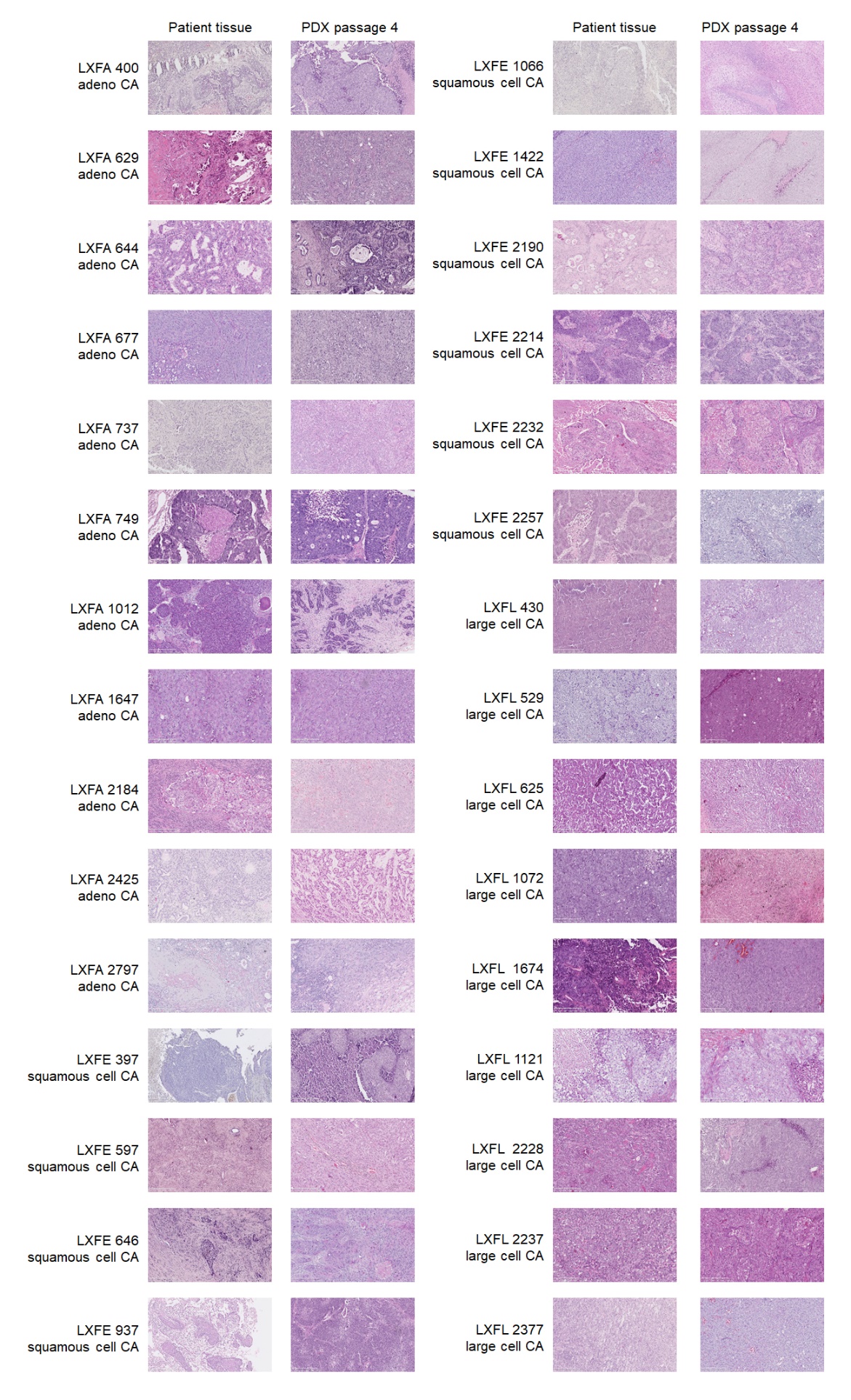

Supplement: Supplementary file 1 — Additional file 1: Supplemental Figure S1. Histological assessment of patient samples and PDX models. Histological features of the selected NSCLC PDX and corresponding patient tissue. H&E stains were prepared from FFPE samples of donor patient tissue as well as the fourth passage of PDX derived thereof. Whole slides were scanned and 10x magnification jpegs extracted of the scans (scale bar included). Supplemental Figure S2. EGFR gene-expression levels correlate with Erlotinib response. EGFR gene-expression measured in the microarray HGU133 (left graph) or by RNA-sequencing (right graph) plotted against the relative tumor size of Erlotinib-treated versus control ZTX models (low values indicate stronger response to Erlotinib). Blue dashed regression lines indicate logarithmic regressions based on the values within the blue dashed circles. [file 13046_2022_2280_MOESM1_ESM.zip › 13046_2022_2280_Fig7_Print.jpeg]

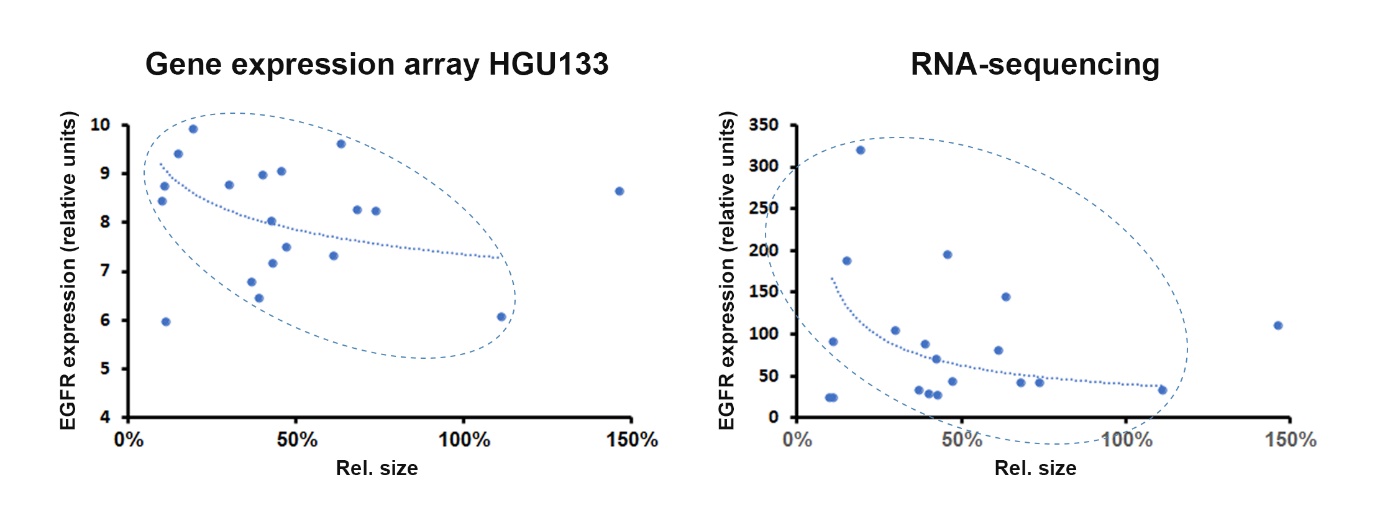

Supplement: Supplementary file 1 — Additional file 1: Supplemental Figure S1. Histological assessment of patient samples and PDX models. Histological features of the selected NSCLC PDX and corresponding patient tissue. H&E stains were prepared from FFPE samples of donor patient tissue as well as the fourth passage of PDX derived thereof. Whole slides were scanned and 10x magnification jpegs extracted of the scans (scale bar included). Supplemental Figure S2. EGFR gene-expression levels correlate with Erlotinib response. EGFR gene-expression measured in the microarray HGU133 (left graph) or by RNA-sequencing (right graph) plotted against the relative tumor size of Erlotinib-treated versus control ZTX models (low values indicate stronger response to Erlotinib). Blue dashed regression lines indicate logarithmic regressions based on the values within the blue dashed circles. [file 13046_2022_2280_MOESM1_ESM.zip › 13046_2022_2280_Fig8_Print.jpeg]
